# The Metal Oxidation State in Cu, CuO, and Cu_2_O Nanoparticles Plays a Key Role in Toxicity to Sea Urchin *Arbacia lixula*, *Paracentrotus lividus,* and *Sphaerechinus granularis* Embryos

**DOI:** 10.3390/toxics13060469

**Published:** 2025-06-01

**Authors:** Ivana Čarapar, Lara Jurković, Dijana Pavičić-Hamer, Andrej Jaklin, Maja Dutour Sikirić, Bojan Hamer, Daniel Mark Lyons

**Affiliations:** 1 Center for Marine Research, Ruđer Bošković Institute, G. Paliaga 5, 52210 Rovinj, Croatia; 2 Division of Physical Chemistry, Ruđer Bošković Institute, Bijenička cesta 54, 10000 Zagreb, Croatia

**Keywords:** antifouling, copper, developmental defect, embryogenesis, marine, paint, skeletogenesis

## Abstract

Copper-based nanoparticles (as Cu_2_O) are a key component in marine antifouling paints and, as coatings degrade, release nanoparticles that can affect a wide range of non-target organisms. This study investigates the impact of Cu_2_O nanoparticles on the early development of urchins *Arbacia lixula*, *Paracentrotus lividus* and *Sphaerechinus granularis*, and benchmarks their toxicity against similarly sized Cu and CuO nanoparticles and ionic copper. Concentration-dependent toxicity was noted for all forms of copper at concentrations in the 1 to 5000 µg L^−1^ range. EC_50_ values after Cu_2_O exposure indicated that *A. lixula* (99 µg L^−1^) was generally more sensitive than the other two species, with EC_50_ values of 371 µg L^−1^ and 606 µg L^−1^ noted for *S. granularis* and *P. lividus*, respectively. The same trend across species was noted for both Cu and CuO, although these nanoparticles generally showed higher EC_50_ values, indicating lower toxicity compared to Cu_2_O. LC_50_ values qualitatively parallel the corresponding EC_50_ values, with Cu_2_O consistently the most toxic, while Cu was less harmful, and CuO did not reach LC_50_ at any concentration. Again, greatest lethality was noted in *A. lixula*. While copper ion release from Cu was much greater than from CuO and Cu_2_O, the latter showed similar or greater toxicity to developing embryos compared to Cu. This indicates that copper ions are not the sole driver of toxicity of Cu_2_O, but there may also be a contribution derived from Cu_2_O redox activity within cells or at membranes that negatively impact oxidative stress defence mechanisms and metabolic pathways.

## 1. Introduction

Copper, an essential element for organisms, is an economically important metal that has found use in a wide range of industrial applications due to its specific electrical, magnetic, and thermal properties. In addition, copper shows favourable characteristics as an antimicrobial agent and, thus, is increasingly used in the wood protection and textile industry due to its antibacterial and antifungal properties such as the ability to generate reactive oxygen species and disrupt cellular membranes in microbes [1,2].

Moreover, copper has become one of the key components in ship hull antifouling paints due to its toxicity to attaching organisms such as polychaetes, algae, and barnacles, which has important commercial implications in terms of reducing drag for enhanced speed and fuel economy in the shipping industry [3,4].

However, over time, hull coatings degrade, and copper-containing biocides may leach into surrounding waters, presenting a threat to non-target organisms [5,6,7]. Indeed, copper concentrations measured at locations such as vessel maintenance yards and marinas are very high and most likely derive from antifouling coatings [8]. Increasingly, with a view of maintaining copper’s efficiency while avoiding significant leaching from the coatings, attention has turned towards encapsulating copper in nanoparticulate form in biocides for hulls [9]. With significant increases in the use of copper-based nanomaterials expected over the coming decade, including in the paints and coatings market [10], the likely quantities of these released into the aquatic environment grows in parallel.

Copper’s diverse modes of toxicity are typically ascribed to the action of Cu^2+^ ions and include, for example, its affinity for binding to histidine, cysteine, and methionine amino acids which impacts protein function [11], binding to sulphydryl groups of proteins and the depletion of antioxidant glutathione [12], and oxidative damage to DNA [13]. Thus, for copper-based nanoparticles, the release of metal ions is thought to be the main driver of toxicity, and negative effects from such nanoparticles have been demonstrated in a wide range of aquatic organisms such as decreased viability and photosynthesis inhibition in microalgae [14], the inhibition of fertilisation ability in sea urchin sperm [15], and the induction of oxidative stress in bivalve haemocytes [16].

The sea urchin, a marine invertebrate, has found wide use in toxicity testing, particularly as urchin embryos provide a number of easily accessible endpoints, including spermiotoxicity and transmissible damage to offspring, anomalous development and skeletogenesis, and cytogenetic anomalies during embryonic development [17,18].

The impact of ionic copper has already been shown in embryos of urchin *Paracentrotus lividus*, where exposure to increasing concentrations of Cu^2+^ resulted in a decrease in the number of embryos reaching the normal plutei larval stage [19], and caused malformations during skeletogenesis such as incomplete or missing skeletal rods, fused arms, and separated or crossed tips [20]. Furthermore, the treatment of sperm with Cu^2+^ similarly showed concentration-dependent effects with decreasing fertilisation success as the copper concentration increased [21]. CuO nanoparticles also showed toxicity to *P. lividus* sperm through the increased generation of reactive oxygen species, lipid peroxidation, DNA fragmentation, and decrease in mitochondrial membrane potential [22], consequently hindering or blocking fertilisation ability [15]. In terms of effects on the zygotes and embryos of sea urchin *Lytechinus pictus*, CuO has been shown to cause concentration-dependent ATP-binding cassette (ABC) transporter inhibition [23], reduction in total antioxidant capacity [24], and developmental defects and growth retardation [25]. These data clearly indicate that copper-based nanoparticles impact a wide range of cellular processes and represent a threat to biota in the aquatic environment.

While relatively few published studies report on the impact of exposure to copper-based nanoparticles in sea urchins, primarily focusing on Cu and CuO nanoparticles, there is a significant knowledge gap regarding the potential toxicity of Cu_2_O nanoparticles in this model organism. This is important as Cu_2_O nanoparticles are a primary constituent of marine antifouling paints and due to leaching may represent a threat to non-target organisms [26,27]. Thus, this study addresses the effects of Cu_2_O nanoparticles on the early life stages and embryonic growth of sea urchin larvae, and is benchmarked against Cu and CuO nanoparticles and ionic copper. Furthermore, the work herein also aimed to define whether different species of sea urchin are equally sensitive to these various sources of copper.

## 2. Materials and Methods

### 2.1. Nanoparticles and Chemicals

Similarly sized copper-based nanoparticles (Cu, 40–60 nm; CuO, <50 nm) were purchased from Sigma-Aldrich (St. Louis, MO, USA), while Cu_2_O (80 nm) was acquired from Nanografi (Ankara, Turkey); copper sulphate pentahydrate was obtained from Kemika (Zagreb, Croatia), while potassium chromium (III) sulphate dodecahydrate and RotiQuant Coomassie dye solution were purchased from Carl Roth (Karlsruhe, Germany). Ultrapure water (18.2 MΩ⸱cm) was provided by a TKA GenPur system (TKA Wasseraufbereitungssysteme GmbH, Niederelbert, Germany), while filtered seawater was prepared by filtering natural seawater though a 0.2 μm mesh cellulose nitrate filter (Whatman, Little Chalfont, UK).

### 2.2. Determination of Nanoparticle Colloidal Stability

Particle size distributions for nanoparticles in ultrapure water and artificial seawater were determined by dynamic light scattering (DLS) using Malvern-Panalytical Zetasizer Nano ZS (Malvern, UK) equipped with a green laser (532 nm). The intensity of scattered light was measured at 173°. Hydrodynamic diameters (*d*_H_) were obtained as the value at peak maximum of the size volume distribution function. Samples were measured 10 times at 25 °C, and the data were processed on Zetasizer v6.32 software.

### 2.3. Nanoparticle Dissolution

Dispersions of Cu, CuO, and Cu_2_O nanoparticles (1 mg-Cu mL^−1^) in ultrapure water and in filtered seawater were prepared every 12 h for 4 days, upon which all dispersions were filtered through Amicon Ultra 3 kDa MWCO centrifugal filters (Merck KGaA, Darmstadt, Germany). This gave a sequence of filtrates from dispersions that had contained nanoparticles for various time periods ranging from 12 h to 96 h. The copper ion concentration in the filtrates was measured by a cupric ion selective electrode (Mettler Toledo, Columbus, OH, USA) to 96 h.

### 2.4. Embryotoxicity Assay

Mediterranean urchins *P. lividus*, *Arbacia lixula* and *Sphaerechinus granularis* were hand collected by divers along the Adriatic coast near Rovinj, Croatia (45°06′30″ N, 13°36′40″ E) during spring (April–May) and autumn (October–November). The urchins were visually inspected and did not display any obvious signs of injury or disease [28] which would present a confounding variable among individuals that would affect the reliability and validity of the experimental results. After collection, urchins were held in flow-through aquaria with running seawater until experiments were carried out, which were always within 5 days of collection. The running seawater was pumped approximately 50 m from the shore at a depth of 10 m, and the location did not have any industrial facilities in the vicinity, indicating low potential for urchin exposure to copper-based contaminants in the flow-through aquaria.

Gametes were obtained by injecting 1 mL potassium chloride (0.5 M) through the peristomal membrane of the sea urchin followed by gentle shaking [29], with eggs from inverted females released into filtered seawater and sperm collected ‘dry’ from upright males by Pasteur pipette. After collecting the gametes, the appearance and motility of the eggs and sperm, respectively, were examined using a light microscope. Eggs that were round without any sign of deformity and sperm that were highly motile were used for subsequent experiments. Fertilisation was carried out in glass beakers by mixing the eggs from each of three females with the sperm from each of two males such that there were 6 permutations overall. The concentration of eggs was ~1000 egg mL^−1^, while the sperm had a final dilution of ×20,000. After 10 min. and periodic stirring, an aliquot from each beaker was examined under a light microscope to confirm elevation of the fertilisation membrane around the eggs. The experiment was continued if fertilisation success was ≥90%.

The required quantity of CuSO_4_ and Cu, CuO, or Cu_2_O nanoparticles (100 μL aliquots of the respective dispersions in ultrapure water), to give final copper concentrations in the range 1–5000 μg L^−1^_,_ were placed in the wells of 10 mL polystyrene 6-well plates, to which was then sequentially added 9 mL filtered seawater and 1 mL fertilised egg suspension (2 h post-fertilisation) from each of the six beakers to the respective well of the multi-well plate. Negative controls were prepared in the same way, except that no toxicant was added to the wells (rather, 100 μL ultrapure water was added instead of the toxicant). The zygotes were held in darkness (to avoid any effect of ambient light on the redox behaviour of the nanoparticles) at 18 °C until they reached the pluteus stage (72 h for *P. lividus* and *A. lixula*; 96 h for *S. granularis*), upon which the plutei were immobilised by the addition of 100 μL chromium sulphate (10 mM), and the first 100 plutei larva in each well were scored among three categories: (i) normally developed, (ii) developmental defects and delayed development, and (iii) arrested development or dead embryos.

### 2.5. Statistical Analysis

Six replicates were carried out in the embryotoxicity assay for each concentration, for each type of copper nanoparticle or copper sulphate, and for each urchin species. Data were tested for normality by the Shapiro–Wilk test and homogeneity of variance by Levene’s test, followed by either parametric ANOVA, with significance differences determined by the Tukey post hoc test, or by non-parametric Kruskal–Wallis ANOVA followed by the Mann–Whitney U-test. Processing of data was carried out using Origin 9 (OriginLab Corporation, Northampton, MA, USA) software. The number of normally developed urchin embryos, or number of dead embryos, after 72 h (*A. lixula, P. lividus*) or 96 h (*S. granularis*) exposure were recorded as a function of copper concentration to determine the half maximal effect concentrations (EC_50_) and median lethal concentrations (LC_50_), respectively. Data were fitted with a dose–response model of form y = A1 + (A2 − A1)/(1 + 10 ^(logEC50-x)*p*^), where A1 and A2 are the bottom and top asymptotes and *p* is the variable Hill slope. A linear regression model was used to estimate EC_50_ and/or LC_50_ values for Cu^2+^ where the bottom and/or top plateaus were insufficient to allow for the use of a standard dose–response model.

## 3. Results

**Figure 1:**
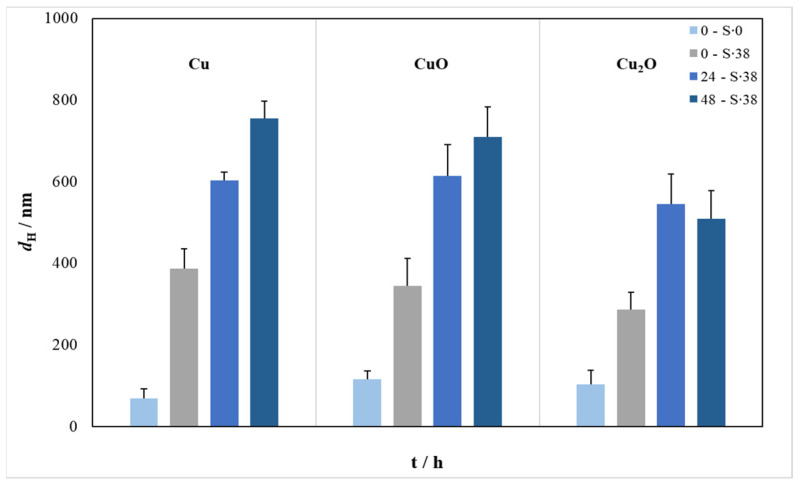
Temporal change in hydrodynamic diameters (*d*_H_) of Cu, CuO, and Cu_2_O nanoparticles (10 mg L^−1^) over 48 h in ultrapure (S‧0) and in artificial seawater (S‧38).

The hydrodynamic diameter (*d*_H_) of the nanoparticles dispersed in ultrapure water and in artificial seawater was determined immediately after preparation, and after 24 h and 48 h (Figure 1).

In ultrapure water, *d*_H_ remained constant for the nanoparticles over time, indicating good colloidal stability. However, in artificial seawater, *d*_H_ immediately increased from about 50 nm to 300–400 nm indicating the rapid formation of agglomerates. After 24 h, the value of *d*_H_ increased to about 600 nm for Cu and CuO, while a value of 550 nm was observed for Cu_2_O, indicating the continued growth of agglomerates over time. After 48 h, *d*_H_ had further increased for Cu and CuO to about 700 nm, while for Cu_2_O there was a slight decrease in the size of the agglomerates, which could suggest that relatively stable agglomerates were already formed after 24 h. During repeated measurements, smaller sized agglomerates were occasionally measured, but they typically comprised less than 5% of the total particles present.

**Figure 2:**
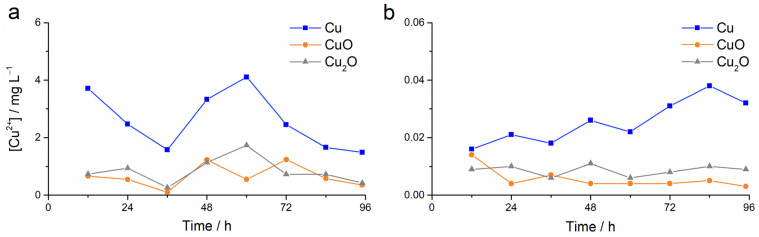
Cupric ion concentration in Cu, CuO, and Cu_2_O dispersions over 96 h in (**a**) ultrapure water and (**b**) filtered seawater.

The presence of cupric (Cu^2+^) ions from nanoparticles dispersed in ultrapure water and filtered seawater was determined by ion selective electrode over 96 h. The seawater in the flow-through acclimation aquaria as well as the filtered seawater used for the experiments did not show the presence of cupric ions (i.e., below the limit of detection of the electrode). From an initial concentration of 1000 mg L^−1^, it was found that the average concentration of released ions from Cu (2.60 ± 1.02 μg L^−1^) in ultrapure water was about 3 times greater than that released from CuO and Cu_2_O (0.65 ± 0.40 and 0.83 ± 0.45 μg L^−1^, respectively), with nanoparticles of both copper oxides releasing similar quantities of ionic copper (Figure 2a). A comparable case was found for filtered seawater, although the average concentrations of cupric ions over 96 h were two orders of magnitude lower than in freshwater (0.026 ± 0.008 μg L^−1^ for Cu, and 0.006 ± 0.004 and 0.009 ± 0.002 μg L^−1^ for CuO and Cu_2_O, respectively; Figure 2b).

**Figure 3:**
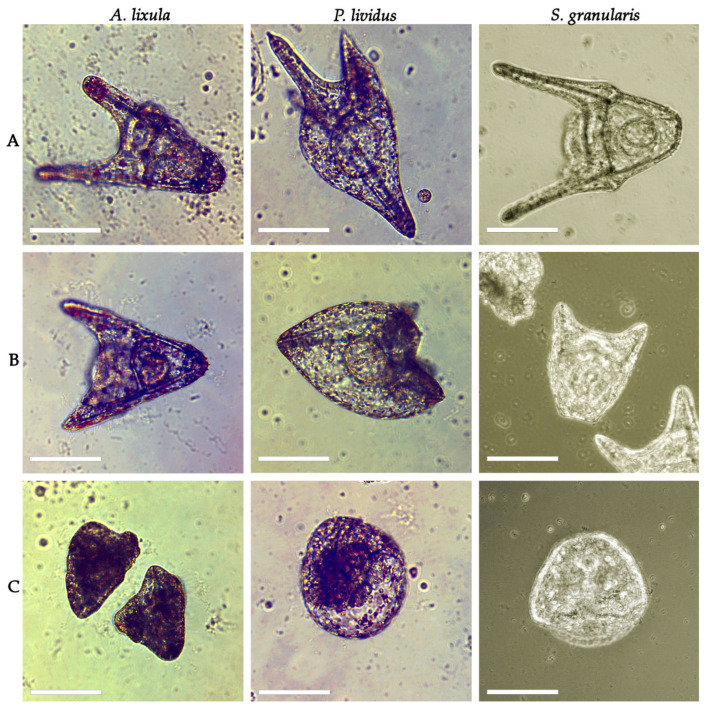
Scoring of embryos and plutei larvae of *A. lixula*, *P. lividus,* and *S. granularis* after exposure to copper in ionic or nanoparticulate form as (**A**) normally developed, (**B**) developmentally delayed, and (**C**) undeveloped or dead. Scale bar = 100 µm.

Embryos and plutei larvae of the three species of urchin were categorised as normally developed, developmentally delayed and defective, and undeveloped embryos based on their visual appearance (Figure 3).

**Figure 4:**
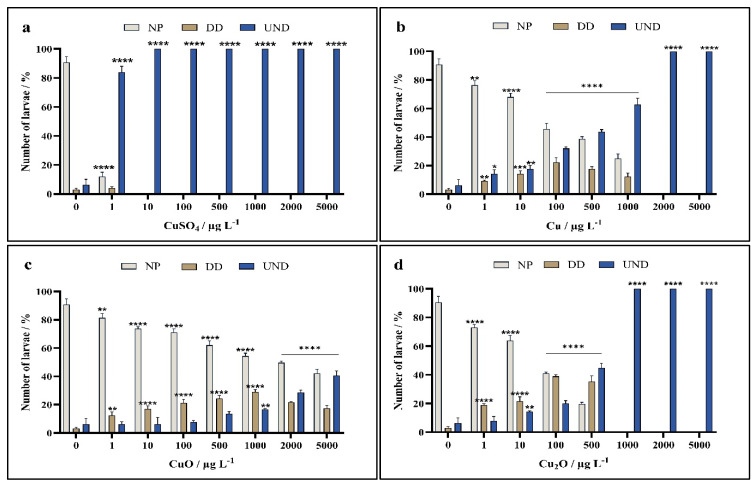
Percentage of normally developed (NP), delayed development and developmental defect, (DD) and undeveloped embryos (UND) of *A. lixula* after 72 h exposure to (**a**) CuSO_4_, and nanoparticles of (**b**) Cu, (**c**) CuO, and (**d**) Cu_2_O. Statistical significance compared to the control indicated as * *p* < 0.05, ** *p* < 0.01, *** *p* < 0.001, and **** *p* < 0.0001.

Zygotes of *A. lixula* exposed to different forms of copper shortly after fertilisation showed clear concentration-dependent trends, with CuSO_4_ showing greater negative effects than for those exposed to nanoparticles. In particular, significant toxicity was noted even at the lowest concentration of 1 µg L^−1^, where the percentage of normal plutei decreased to 16% compared to the non-treated controls with 94% normal plutei. At all other copper concentrations, there was a complete absence of live embryos and larvae (Figure 4a).

For zygotes exposed to Cu nanoparticles, a trend in decreasing number of normal plutei with increasing nanoparticle concentration was noted, with a significant reduction in normal plutei already noted at the lowest copper concentration of 1 µg L^−1^, and only about 50% normal plutei remaining after treatment with 100 µg L^−1^ nanoparticulate Cu (Figure 4b). Only about 30% plutei remained alive at a copper concentration of 1000 µg L^−1^, while all were dead at higher concentrations.

While significant differences to controls were noted for all concentrations of CuO nanoparticles, the nanoparticles were observed to cause lower toxicity than Cu nanoparticles, with a large proportion of plutei (42%) scored as normal up to the highest tested concentration of 5000 µg L^−1^ (Figure 4c).

In contrast, toxicity effects from exposure to Cu_2_O nanoparticles more closely resembled that of Cu nanoparticles, where significant differences to controls were noted for all concentrations, with only about 40% normally developed plutei observed at a CuO concentration of 100 µg L^−1^ (Figure 4d). At a concentration of 500 µg L^−1^, a preponderance of dead embryos was noted, while only dead embryos were found at all higher concentrations.

**Figure 5:**
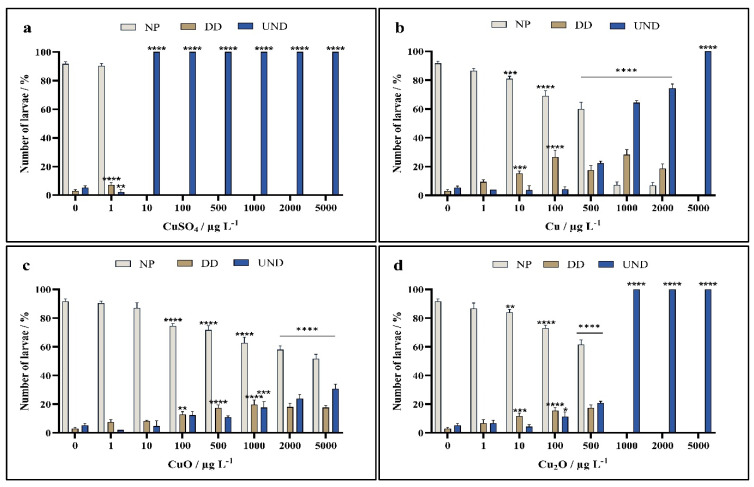
Percentage of normally developed (NP), delayed development and developmental defect (DD), and undeveloped embryos (UND) of *P. lividus* after 72 h exposure to (**a**) CuSO_4_, and nanoparticles of (**b**) Cu, (**c**) CuO, and (**d**) Cu_2_O. Statistical significance compared to the control indicated as * *p* < 0.05, ** *p* < 0.01, *** *p* < 0.001, and **** *p* < 0.0001.

In the case of developing *P. lividus* embryos, similar trends were noted as for *A. lixula*, with zygotes treated with CuSO_4_ showing greater negative effects than for those exposed to nanoparticles. Ionic copper, in the form of copper sulphate, showed little effect on plutei after 72 h exposure at a concentration of 1 μg L^−1^, while 10 μg L^−1^ copper ions resulted in complete developmental arrest at the 4–8 cell stage, i.e., arrest almost immediately after exposure (Figure 5a). Cu nanoparticles showed a milder effect, with most larvae showing normal development up to a concentration of 500 μg L^−1^, although numbers of normal larvae were significantly lower than the control. In parallel, the number of larvae with delayed or arrested development began to significantly increase from copper concentrations of 10 and 500 μg L^−1^, respectively, with more than half showing complete developmental arrest at concentrations from 1000 μg L^−1^ (Figure 5b).

Exposure of zygotes to CuO nanoparticles resulted in a significant reduction in normal embryonic development at copper concentrations of 100 µg L^−1^ and higher, with a concomitant significant increase in number of plutei larvae showing developmental delays and arrest (Figure 5c). However, even at a copper concentration of 5000 µg L^−1^, most of the plutei were normally developed without any obvious deformities, unlike the case for Cu nanoparticles where significant negative effects were noted as much lower copper concentrations.

Similarly to Cu nanoparticles, zygotes exposed to Cu_2_O nanoparticles also showed a significant decrease in normal plutei at a copper concentration of 10 µg L^−1^ with a parallel increase in the number of developmentally delayed plutei and those showing skeletal defects (Figure 5d). The number of normal plutei decreased with increasing copper concentration, with about 60% normal plutei noted for the 500 µg L^−1^ treatment. In contrast to Cu nanoparticles, for Cu_2_O nanoparticles with copper concentrations greater than 500 µg L^−1^ no living embryos or plutei were noted after the 72 h exposure.

**Figure 6:**
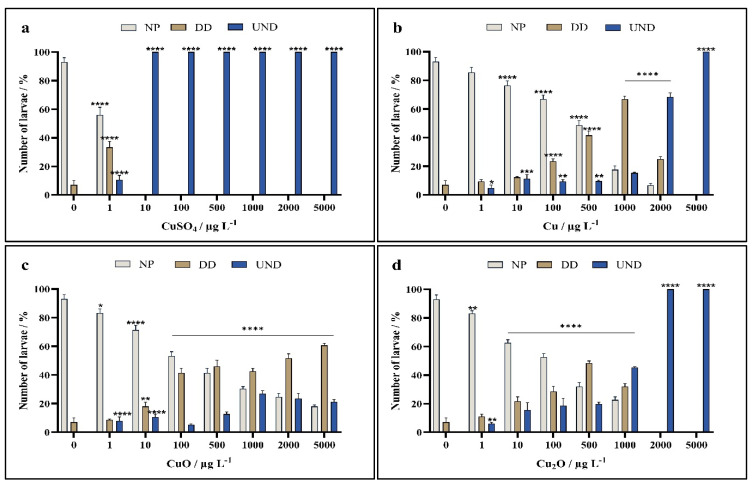
Percentage of normally developed (NP), delayed development and developmental defects (DD) and undeveloped embryos (UND) of *S. granularis* after 96 h exposure to (**a**) CuSO_4_, and nanoparticles of (**b**) Cu, (**c**) CuO, and (**d**) Cu_2_O. Statistical significance compared to the control indicated as * *p* < 0.05, ** *p* < 0.01, *** *p* < 0.001 and **** *p* < 0.0001.

Consistent with data from the other urchins, embryos and plutei of *S. granularis* showed qualitatively similar effects from exposure to ionic and nanoparticulate copper. The greatest negative impact was recorded after the treatment of zygotes with CuSO_4_, where the percentage of normally developed larvae decreased from 90% to about 55% compared to the control after exposure to the lowest Cu^2+^ concentration of 1 µg L^−1^, while at all other concentrations (10–5000 µg L^−1^) all embryos were dead (Figure 6a).

Treatment of zygotes with Cu nanoparticles significantly reduced the development of normal larvae at a concentration of 10 µg L^−1^, with only half the plutei classed as normal after exposure to 500 µg L^−1^ nanoparticulate copper (Figure 6b). At higher Cu nanoparticle concentrations, developmentally delayed plutei (1000 µg L^−1^ Cu) or developmentally arrested embryos (≥2000 µg L^−1^ Cu) dominated.

The effects of treatment with CuO nanoparticles were more pronounced on *S. granularis* zygotes compared to *A. lixula* and *P. lividus*. While significant effects were noted already at a concentration of 1 µg L^−1^, less than 50% of the plutei were normal at concentrations >100 µg L^−1^ while at all higher nanoparticulate copper concentrations most of the remaining plutei showed developmental delays (50–60%), with a smaller number of dead embryos or plutei (20–30%) present (Figure 6c).

Similar behaviour was noted for zygotes exposed to Cu_2_O nanoparticles where increasing copper concentrations resulted in a corresponding decrease in normally developed plutei and a parallel increase in developmentally delayed plutei up to a concentration of 500 µg L^−1^, after which most (1000 µg L^−1^ Cu) or all (≥2000 µg L^−1^ Cu) embryos and plutei were found to be dead (Figure 6d).

Half maximal effect concentrations (EC_50_) were calculated for embryos of the three species of urchin exposed to ionic and nanoparticulate copper, and the values are given in Table 1. *P. lividus* was generally found to be more robust than the other two species, with greater copper concentrations required to achieve a reduction to 50% normal plutei. For example, exposure to Cu_2_O required a concentration of 606 µg L^−1^ to reduce the proportion of normal larvae of *P. lividus* to 50%, while lower copper concentrations of 371 µg L^−1^ and 99 µg L^−1^ were sufficient to achieve the same negative effect in *S. granularis* and *A. lixula respectively.* Conversely, *A. lixula* was typically the most sensitive species, with much lower copper concentrations necessary to achieve EC_50_, for example, exposure to 88 µg L^−1^ Cu nanoparticles in contrast to 496 µg L^−1^ and 617 µg L^−1^ for *P. lividus* and *S. granularis*, respectively. In terms of nanoparticle toxicity, Cu_2_O in most cases caused a greater negative effect than Cu (similar values in *A. lixula*) while CuO was generally found to have the mildest effects (except in *S. granularis*).

Median lethal concentrations (LC_50_) were also calculated for the three urchin species based on the number of dead zygotes/embryos/plutei after 72 h or 96 h exposure to four sources of copper (Table 2). The values qualitatively parallel the corresponding EC_50_ values, indicating that *A. lixula* is the most sensitive species to copper. However, *S. granularis* indicated higher LC_50_ values than *P. lividus*, unlike the case for EC_50_ where the order of species robustness was reversed. Moreover, of the nanoparticles tested, Cu_2_O was consistently found to cause the greatest toxicity, while Cu was less harmful, and CuO did not reach LC_50_ at any of the tested concentrations.

## 4. Discussion

Many organisms are known to be susceptible to copper toxicity, and reports in the literature have typically compared and contrasted the effects of Cu and CuO nanoparticles. However, much more rarely have Cu_2_O nanoparticles been investigated, which is surprising given this material’s wide range of commercial uses, particularly as a biocide. In this work, the effects of Cu_2_O nanoparticles, as well as comparison with ionic and nanoparticulate Cu and CuO, on embryogenesis and larval development in several species of sea urchin were studied as a function of concentration.

Irrespective of the form or oxidation state of copper, clear concentration-dependent trends were noted. In particular, with increasing copper concentration, the number of normally developed plutei decreased in parallel with an increasing number of plutei showing delayed or anomalous development, arrested development at earlier development phases or dead zygotes and embryos. Indeed, significant negative effects (compared to non-treated controls) were oftentimes noted at the lowest copper concentrations used, indicating very high sensitivity of urchin zygotes, embryos, and plutei to the presence of copper. The toxicity of copper nanoparticles has typically been ascribed to Cu^2+^ ions released from the particle surface, with the greater surface areas (per unit mass) of smaller nanoparticles showing more rapid release of Cu^2+^ ions than larger particles. A study on copper nanoparticle (40, 60, 80 nm) size effects on dorsal root ganglion neurons of Sprague Dawley rats clearly differentiated the increased toxicity of copper nanoparticles with decreasing size [30]. In sea urchins, there are relatively few reports on the effects of copper-based nanoparticles on embryonal development. It was reported that 12 nm CuO nanoparticles (80–200 nm aggregates in seawater) disrupt cholinergic and serotonergic pathways of the nervous system in *A. lixula, for example* through dose-dependent decreased levels of choline and *N*-acetyl serotonin, as well as giving rise to developmental anomalies during skeletogenesis, with EC_50_ values lying in the 10–20 μg L^−1^ range [31,32]. Moreover, the analysis of gene expression found that not only was there a concentration-dependent increase in glycine, a constituent of matrix proteins involved in biomineralisation but that expression of genes related to antioxidant and cytoprotective activities (catalase, superoxide dismutase, and metallothionein) had decreased after exposure to the CuO nanoparticles [33]. The corresponding EC_50_ values in the present work are far higher for CuO (1629 μg L^−1^) in *A. lixula*, but it should be noted that the nanoparticle size is also much larger (about 50 nm) as well as the size of the agglomerates (about 700 nm) in the seawater medium, indicating that reduced nanoparticle dissolution and ion release may be responsible for the difference in results. Unfortunately, it is not possible to benchmark results based on Cu^2+^ ion effects as those works did not report EC_50_ values for CuSO_4_ as positive controls.

Similar studies were carried out on the effects of CuO on sea urchin *L. pictus* using 10 nm synthesised and 50 nm commercially obtained nanoparticles [24]. Again, the smaller sized nanoparticles showed greater dissolution (2.5%) than the larger particles (0.74%) with a corresponding effect on developmental anomalies, with EC_50_ values of 450 and 5395 μg L^−1^ noted, respectively. These larger commercially obtained nanoparticles showed very little effect on embryonic growth at environmentally relevant concentrations, which is broadly in line with the data from the present work where 50 nm particles showed very high EC_50_ values (with the exception of *S. granularis*) and LC_50_ was not attained after treating zygotes at the highest concentrations of 5000 μg L^−1^. It should be noted that their EC_50_ value of 32 μg L^−1^ for Cu^2+^ is somewhat higher than that of the present work (0.5 μg L^−1^) although this may be related to the different species and physico-chemical properties of the nanoparticles used—for comparison, the EC_50_ values for Cu^2+^ causing a decrease in the number of normal *P. lividus* plutei or size impairment were recently reported as in the range 24–36 μg L^−1^ [34], 67 μg L^−1^ [19], and 380 μg L^−1^ [35]. Not only was exposure to nanoparticulate CuO soon after fertilisation found to cause significant developmental defects but also to result in the inhibition of ABC efflux transporters [23] and a decrease in total antioxidant capacity [25], which is in line with alterations in gene expression related to antioxidant pathways [33]. Interestingly, greater accumulation of nanoparticulate copper in the embryos compared to soluble copper (Cu^2+^) was shown, which may be one of the key factors in the deleterious effects recorded [25]. Indeed, a similar effect was reported for 10 nm silver nanoparticles where greater bioavailability and easier internalisation caused higher toxicity in *Escherichia coli* compared to Ag^+^ ions alone [36].

It is important to mention here that high levels of agglomeration are the likely reason why our applied nanoparticulate copper concentrations >1000 μg L^−1^ did not result in a concentration-dependent reduction in normal plutei and parallel increase in dead plutei.

In the present work, while the nanoparticles were of broadly similar size (considering nominal size and size distributions), with Cu and CuO having diameters of about 50 nm and Cu_2_O of 80 nm, the size difference and different chemical content meant that clearly determining size-dependent toxicity is not readily achievable. However, as Cu^2+^ ions are considered the primary driver of toxicity, a comparison of cupric ion concentrations in the exposure medium (filtered seawater) may provide a useful indicator of potential differences in toxicity. The speciation of copper nanoparticles in aqueous media at acidic pH encompasses the Cu^2+^ ionic form with a small quantity of Cu(OH)^+^ present (at pH values down to about pH 6). At the high ionic strength of seawater used herein (about 0.7 M) and at pH 8, dissolution of nanoparticles is suppressed. Specifically, with increasing ionic strength, charge shielding and disruption of the electric double layer drives agglomeration of the nanoparticles which disfavours dissolution. Agglomerates of Cu (100–1000 nm) and CuO (20–100 nm) nanoparticles in seawater have been reported with diameters of about 5 μm and 450 nm, respectively [37], while in the present work a gradual increase in agglomerate hydrodynamic diameter over 48 h to about 800 nm (Cu) and 700 nm (CuO) was noted. In comparison, Cu_2_O agglomerates were found to have much smaller diameters of about 450 nm. Any metal ions released from the nanoparticles and agglomerates will generally form a range of complexes such as CuCO_3_, CuHCO_3_^+^, Cu(OH)^+^, Cu(OH)_2_, CuCl^+^, CuCl_2_, CuSO_4_ and CuOHCl, while concentrations of free Cu^2+^ remain extremely low (about 1% of total copper) [38,39]. However, the presence of dissolved organic matter in seawater, such as the extracellular polymeric substances secreted by microorganisms that can form a layer around nanoparticles, counteracts their tendency to agglomerate and subsequently enables greater metal release from the particle surfaces [40,41]. Herein, Cu_2_O nanoparticle agglomerate sizes were much smaller than those of Cu and CuO nanoparticles, indicating a higher degree of stabilisation that may have enabled greater ion release and hence toxicity to urchin zygotes, embryos, and plutei. However, while nanoparticle stabilisation would be expected to increase copper nanoparticle toxicity, it should be noted that dissolved organic matter can chelate and sequester metal cations. Indeed, measurement of cupric ion concentrations by an ion-selective electrode showed similar values for both CuO and Cu_2_O, even though the latter displayed much smaller agglomerate sizes. Furthermore, dissolved organic matter can provide a protective effect, as has been shown both for fulvic and humic acids, where the dissolved organic matter reduced the toxicity of ionic copper in developing embryos of *P. lividus*, for example, EC_50_ values of 41.1 and 32.9 μg L^−1^ reported for copper with and without humic acid, respectively [42,43].

As the free cupric ion concentrations in CuO and Cu_2_O dispersions were similar over several days (about 0.1% and 0.001% of the initial nanoparticulate copper in ultrapure water and filtered seawater, respectively), and 2–3 times less than that of the Cu dispersion, it may be expected that EC_50_ and LC_50_ values would reflect that difference. However, the Cu_2_O exposure showed similar toxicity to that of Cu, while exposure of zygotes to CuO had much milder effects and even at very high nanoparticle mass, the LC_50_ level was typically not attained (likely due to sedimentation of very large nanoparticle agglomerates and reduced bioavailability). These results indicate that Cu^2+^ concentration alone may not be the only factor contributing to nanoparticle toxicity. It has already been reported that the physical interaction of nanomaterials with cell membranes can cause significant disruption and even penetration [44], which may lead to potentially greater or more rapid cellular uptake of these materials. Thus, the exposure of urchin zygotes 2 h post-fertilisation, when in a phase of rapid cell division, to copper nanoparticles may have resulted in membrane–particle interactions that lead to greater negative outcomes, including development delays and arrest. In addition, such physical interactions that may support the cellular uptake of particles (or copper ions) provide a mechanism where Cu^2+^ are generated directly within the cell by release from nanoparticles, leading to a cascade of negative effects on cellular function, for example, through DNA damage and apoptosis [45]. Considering that the Cu_2_O (80 nm) nanoparticles are nominally larger than the Cu (<50 nm) nanoparticles, it may be expected that the latter would be more readily taken up and, along with greater ion release, the Cu nanoparticles should have resulted in significantly greater deleterious outcomes for the developing embryos. However, as EC_50_ and LC_50_ values for Cu and CuO nanoparticles are similar for the individual species of urchin, other modes are necessary to account for the toxicity of the Cu_2_O nanoparticles. Dissolution of Cu_2_O nanoparticles, for example, in the low pH environment in cellular compartments such as endosomes and lysosomes (pH 4.5–6.5), supports the release of Cu^+^ ions which may persist in those organelles [46] or be oxidised to Cu^2+^ by Fenton-like reactions, for example, by hydrogen peroxide, resulting in the generation of hydroxyl radicals which contribute to cellular oxidative stress such as lipid peroxidation and disruption to enzymatic activity [47,48,49]. This may be a significant contributing factor as to why Cu_2_O nanoparticles induced far greater toxicity in developing embryos than the CuO nanoparticles, even though measured cupric ion concentrations were nearly the same.

Interestingly, while general trends of copper toxicity were observed, not all species of urchin demonstrated the same sensitivity at given concentrations of copper. Overall, *A. lixula* was shown to be the most sensitive while *P. lividus* was the most robust in terms of delayed development based on half maximal effect concentrations (EC_50_). As early embryonic development in the different urchin species is very similar, the reason for the different impacts of nanoparticles in the three species may be related to differential modulation of gene expression profiles upon exposure to toxicants. Changes in the regulation of oxidative-stress related genes in *A. lixula* after exposure to CuO nanoparticles [33] or changes in metallothionein expression in *P. lividus* after exposure to metals such as copper [50] may indicate the primary source of species-specific sensitivity to toxicants, which is derived from the need to maintain a balance between copper homeostasis and copper detoxification pathways. Indeed, a comparative analysis of gene (dys)regulation and protein expression profiles for the three urchin species exposed under the same experimental conditions would likely provide confirmation of the basis for species showing more or less robust behaviour in the presence of particular toxicants. Species-specific sensitivity found herein is consistent with data presented for three species of urchin treated with silver nanoparticles [51]. A recent report on the effect of silver nanoparticle size on embryonic development in *A. lixula* and *P. lividus* provided contrasting data, where EC_50_ values for smaller nanoparticles (10, 20, 40 nm) were in many cases similar for both species, while for larger nanoparticles (60, 100 nm), *P. lividus* had lower calculated EC_50_ values indicating greater sensitivity [52]. Interestingly, the provided EC_50_ values for 60 nm silver nanoparticles of 846 and 360 μg L^−1^ for *A. lixula* and *P. lividus*, respectively, are larger than the corresponding values reported by Burić et al. [51]. However, the major difference between those reports is that the nanoparticles used in the earlier work were synthesised in-house and had a less uniform spherical shape compared to the commercially provided nanoparticles used in the more recent work. This suggests that particles with a less ‘smooth’ surface with more edges and vertices are more toxic to urchin embryos and may be related to more rapid oxidative dissolution of the silver nanoparticles at defect sites at the surface. Shape-specific effects for silver nanoparticles have also been noted with other aquatic organisms where triangular nano-plates, with more crystal defects, were found to show greater toxicity to *Danio rerio* and a gill cell line of *Oncorhynchus mykiss* than spherical particles and nanowires [53]. This seems to be a broadly common effect, as reports on other nanomaterials support the conclusion that particle forms that possess more edges and defects induce greater toxicity. In particular, a study on nanoparticles (<50 nm) and nanosheets (250–1000 nm^2^ × 15 nm thick) of CuO found greater anti-microbial activity towards *E. coli* for the nanosheets than for the nanoparticles [54].

The broader issue of spatial and temporal variability of spawning populations should also be considered, with significant variations in EC_50_ values noted for embryos derived from adult urchins harvested from different locations and at different times of the year [55], as well as the previous exposure profile of the adult urchins to toxicants [56]. In the present study, urchins were collected near a site where copper concentrations in sediment were found to be about 13 mg/kg d.w. (below regulatory limits), although this represents long-term accumulation of the metal rather than an acute input, so the copper concentration in the water column and on hard rocky substrates where the urchins prefer to live is likely to be far lower than this value [57]. Moreover, despite the possibility of organisms being exposed to environmental copper, the experiment is a comparative study of urchins collected from the same location, so untreated (control) and treated (Cu, CuO, and Cu_2_O NPs) embryos all derive from adult urchins from the same area and would be expected to have very similar environmental exposure to background copper. Overall, care should be taken when comparing such values among published works should genetic, temporal, and spatial aspects not be adequately taken into account.

## 5. Conclusions

The toxicity of Cu_2_O nanoparticles towards developing embryos of sea urchins was investigated and benchmarked against the impact of similarly sized nanoparticles of Cu and CuO. All nanoparticles and ionic copper showed concentration-dependent toxicity. The Cu_2_O nanoparticles were found to cause more negative effects than the other nanoparticles, resulting in significant developmental delays, skeletal anomalies and lethality. Such effects were noted in three species of evolutionary distant sea urchins, with the greatest damage noted in *A. lixula*, while *P. lividus* and *S. granularis* were more robust. Interestingly, leaching of copper ions from Cu_2_O was much lower than for Cu, even though in cases both nanoparticles showed somewhat similar toxicity during embryogenesis. This may indicate that Cu_2_O redox chemistry can play a role, where the deleterious effects of copper ion-induced oxidative stress may be added to by catalytically driven processes at nanoparticle surfaces. Considering the toxicity of nanoparticulate Cu_2_O and the fact that it is increasingly used in antifouling paints, the scarcity of published data on its effects on non-target organisms clearly indicates that further research efforts are warranted. More broadly, gaining an understanding of species-specific responses and the biophysical interactions at play is key for developing more comprehensive assessments of copper nanoparticle toxicity which could inform regulatory frameworks and environmental safety standards concerning the use and disposal of these engineered nanoparticles.

## Figures and Tables

**Table 1:** Half maximal effect concentrations (EC_50_) for urchin embryos exposed to ionic (Cu^2+^) and nanoparticulate (Cu, CuO, Cu_2_O) copper for 72 h (*A. lixula*, *P. lividus*) or 96 h (*S. granularis*). Values are given as the mean ± standard error; *nd* = not defined, as <50% embryos show a defined effect from copper exposure. Values in italics are derived from a linear regression model.

EC_50_	*A. lixula*	*P. lividus*	*S. granularis*
	μg L^−1^	μg L^−1^	μg L^−1^
Cu^2+^	*0.51*	*5.06*	*1.92*
Cu	88 ± 16	617 ± 89	496 ± 116
CuO	1629 ± 548	*nd*	91 ± 47
Cu_2_O	99 ± 39	606 ± 90	371 ± 169

**Table 2:** Median lethal concentrations (LC_50_) for urchin embryos exposed to ionic (Cu^2+^) and nanoparticulate (Cu, CuO, Cu_2_O) copper for 72 h (*A. lixula*, *P. lividus*) or 96 h (*S. granularis*). Values are given as the mean ± standard error; *nd* = not defined, as <50% embryos showed lethality from copper exposure. Values in italics are derived from a linear regression model.

LC_50_	*A. lixula*	*P. lividus*	*S. granularis*
	μg L^−1^	μg L^−1^	μg L^−1^
Cu^2+^	*0.56*	*5.39*	*4.90*
Cu	695 ± 86	837 ± 17	1764 ± 83
CuO	*nd*	*nd*	*nd*
Cu_2_O	559 ± 40	622 ± 124	1108 ± 115

## Data Availability

All data are reported in this manuscript.

## References

[1] Raffi M., Mehrwan S., Bhatti T.M., Akhter J.I., Hameed A., Yawar W., Ul Hasan M.M. (2010). Investigations into the antibacterial behavior of copper nanoparticles against Escherichia coli. Ann. Microbiol..

[2] Pariona N., Mtz-Enriquez A.I., Sánchez-Rangel D., Carrión G., Paraguay-Delgado F., Rosas-Saito G. (2019). Green-synthesized copper nanoparticles as a potential antifungal against plant pathogens. RSC Adv..

[3] Adeleye A.S., Oranu E.A., Tao M., Keller A.A. (2016). Release and detection of nanosized copper from a commercial antifouling paint. Water Res..

[4] Avelelas F., Martins R., Oliveira T., Maia F., Malheiro E., Soares A.M.V.M., Loureiro S., Tedim J. (2017). Efficacy and ecotoxicity of novel anti-fouling nanomaterials in target and non-target marine species. Mar. Biotechnol..

[5] Amara I., Miled W., Slama R.B., Ladhari N. (2018). Antifouling processes and toxicity effects of antifouling paints on marine environment. A review. Environ. Toxicol. Pharmacol..

[6] Lagerström M., Ytreberg E., Wiklund A.E., Granhag L. (2020). Antifouling paints leach copper in excess—Study of metal release rates and efficacy along a salinity gradient. Water Res..

[7] Miller R.J., Adeleye A.S., Page H.M., Kui L., Lenihan H.S., Keller A.A. (2020). Nano and traditional copper and zinc antifouling coatings: Metal release and impact on marine sessile invertebrate communities. J. Nanopart. Res..

[8] Torres F.G., De-la-Torre G.E. (2021). Environmental pollution with antifouling paint particles: Distribution, ecotoxicology, and sustainable alternatives. Mar. Pollut. Bull..

[9] Loredo-Becerra G.M., Durán-Almendárez A., Calvillo-Anguiano A.K., DeAlba-Montero I., Hernández-Arteaga L.O., Ruiz F. (2022). Waterborne antifouling paints containing nanometric copper and silver against marine *Bacillus* species. Bioinorg. Chem. Appl..

[10] Keller A.A., Ehrens A., Zheng Y., Nowack B. (2023). Developing trends in nanomaterials and their environmental implications. Nat. Nanotechnol..

[11] Camakaris J., Voskoboinik I., Mercer J.F. (1999). Molecular mechanisms of copper homeostasis. Biochem. Biophys. Res. Commun..

[12] Speisky H., Gómez M., Burgos-Bravo F., López-Alarcón C., Jullian C., Olea-Azar C., Aliaga M.E. (2009). Generation of superoxide radicals by copper-glutathione complexes: Redox-consequences associated with their interaction with reduced glutathione. Bioorg. Med. Chem..

[13] Moriwaki H., Osborne M.R., Phillips D.H. (2008). Effects of mixing metal ions on oxidative DNA damage mediated by a Fenton-type reduction. Toxicol. Vitr..

[14] Chebotaryova S.P., Zakharova O.V., Gusev A.A., Baranchikov P.A., Kolesnikov E.A., Yakusheva A.S., Skripnikova E.V., Lobakova E.S., Xu J., Alam M.A. (2023). Assessment of the tolerance of a chlorophyte *Desmodesmus* to CuO-NP for evaluation of the nanopollution bioremediation potential of this microalga. Nanomaterials.

[15] Rotini A., Gallo A., Parlapiano I., Berducci M.T., Boni R., Tosti E., Prato E., Maggi C., Cicero A.M., Migliore L. (2018). Insights into the CuO nanoparticle ecotoxicity with suitable marine model species. Ecotoxicol. Environ. Saf..

[16] Zha S., Rong J., Guan X., Tang Y., Han Y., Liu G. (2019). Immunotoxicity of four nanoparticles to a marine bivalve species, *Tegillarca granosa*. J. Hazard. Mater..

[17] Pagano G., Thomas P., Guida M., Palumbo A., Romano G., Oral R., Trifuoggi M. (2017). Sea urchin bioassays in toxicity testing: II. Sediment evaluation. Expert Opin. Environ. Biol..

[18] Pagano G., Guida M., Trifuoggi M., Thomas P., Palumbo A., Romano G., Oral R. (2017). Sea urchin bioassays in toxicity testing: I. Inorganics, organics, complex mixtures and natural products. Expert Opin. Environ. Biol..

[19] Fernandez N., Beiras R. (2001). Combined toxicity of dissolved mercury with copper, lead and cadmium on embryogenesis and early larval growth of the *Paracentrotus lividus* sea urchin. Ecotoxicology.

[20] Morroni L., Pinsino A., Pellegrini D., Regoli F. (2018). Reversibility of trace metals effects on sea urchin embryonic development. Ecotoxicol. Environ. Saf..

[21] Manzo S., Buono S., Cresimini C. (2008). Predictability of copper, Irgarol, and diuron combined effects on sea urchin *Paracentrotus lividus*. Arch. Environ. Contam. Toxicol..

[22] Gallo A., Manfra L., Boni R., Rotini A., Migliore L., Tosti E. (2018). Cytotoxicity and genotoxicity of CuO nanoparticles in sea urchin spermatozoa through oxidative stress. Environ. Int..

[23] Wu B., Torres-Duarte C., Cole B.J., Cherr G.N. (2015). Copper oxide and zinc oxide nanomaterials act as inhibitors of multidrug resistance transport in sea urchin embryos: Their role as chemosensitizers. Environ. Sci. Technol..

[24] Torres-Duarte C., Adeleye A.S., Pokhrel S., Mädler L., Keller A.A., Cherr G.N. (2016). Developmental effects of two different copper oxide nanomaterials in sea urchin (*Lytechinus pictus*) embryos. Nanotoxicology.

[25] Torres-Duarte C., Ramos-Torres K.M., Rahimoff R., Cherr G.N. (2017). Stage specific effects of soluble copper and copper oxide nanoparticles during sea urchin embryo development and their relation to intracellular copper uptake. Aquat. Toxicol..

[26] Blossom N., Szafranski F., Lotz A. (2018). Use of copper-based antifouling paint: A U.S. regulatory update. CoatingsTech.

[27] Cima F., Varello R. (2023). Potential disruptive effects of copper-based antifouling paints on the biodiversity of coastal macrofouling communities. Environ. Sci. Pollut. Res. Int..

[28] Wang Y.N., Chang Y.Q., Lawrence J.M., Lawrence J.M. (2013). Disease in sea urchins. Sea Urchins: Biology and Ecology. Developments in Aquaculture and Fisheries Science.

[29] Thomas P.J., Oral R., Pagano G., Tez S., Toscanesi M., Ranieri P., Trifuoggi M., Lyons D.M. (2020). Mild toxicity in *Paracentrotus lividus* early life stages may indicate species-specific sensitivity to polystyrene and polymethylmethacrylate microplastics. Mar. Environ. Res..

[30] Prabhu B.M., Ali S.F., Murdock R.C., Hussain S.M., Srivatsan M. (2009). Copper nanoparticles exert size and concentration dependent toxicity on somatosensory neurons of rat. Nanotoxicology.

[31] Maisano M., Cappello T., Catanese E., Vitale V., Natalotto A., Giannetto A., Barreca D., Brunelli E., Mauceri A., Fasulo S. (2015). Developmental abnormalities and neurotoxicological effects of CuO NPs on the black sea urchin *Arbacia lixula* by embryotoxicity assay. Mar. Environ. Res..

[32] Cappello T., Vitale V., Oliva S., Villari V., Mauceri A., Fasulo S., Maisano M. (2017). Alteration of neurotransmission and skeletogenesis in sea urchin *Arbacia lixula* embryos exposed to copper oxide nanoparticles. Comp. Biochem. Physiol. C.

[33] Giannetto A., Cappello T., Oliva S., Parrino V., De Marco G., Fasulo S., Mauceri A., Maisano M. (2018). Copper oxide nanoparticles induce the transcriptional modulation of oxidative stress-related genes in *Arbacia lixula* embryos. Aquat. Toxicol..

[34] Morroni L., Sartori D., Costantini M., Genovesi L., Magliocco T., Ruocco N., Buttino I. (2019). First molecular evidence of the toxicogenetic effects of copper on sea urchin *Paracentrotus lividus* embryo development. Water Res..

[35] Garcia-Velasco N., Carrero J.A., Urionabarrenetxea E., Doni L., Zaldibar B., Izagirre U., Soto M. (2023). Innovative in vivo and in vitro bioassays for the establishment of toxicity thresholds of pollutants in sediment quality assessment using polychaetes and their immune cells. Chemosphere.

[36] Ivask A., Kurvet I., Kasemets K., Blinova I., Aruoja V., Suppi S., Vija H., Käkinen A., Titma T., Heinlaan M. (2014). Size-dependent toxicity of silver nanoparticles to bacteria, yeast, algae, crustaceans and mammalian cells in vitro. PLoS ONE.

[37] Conway J.R., Adeleye A.S., Gardea-Torresdey J., Keller A.A. (2015). Aggregation, dissolution, and transformation of copper nanoparticles in natural waters. Environ. Sci. Technol..

[38] Allen H., Hansen D. (1996). The importance of trace metal speciation to water quality criteria. Water Environ. Res..

[39] Adeleye A.S., Conway J.R., Perez T., Rutten P., Keller A.A. (2014). Influence of extracellular polymeric substances on the long-term fate, dissolution, and speciation of copper-based nanoparticles. Environ. Sci. Technol..

[40] Arnold W.R., Cotsifas J.S., Ogle R.S., DePalma S.G.S., Smith D.S. (2010). A comparison of the copper sensitivity of six invertebrate species in ambient salt water of varying dissolved organic matter concentrations. Environ. Toxicol. Chem..

[41] Zitoun R., Clearwater S.J., Hassler C., Thompson K.J., Albert A., Sander S.G. (2019). Copper toxicity to blue mussel embryos (*Mytilus galloprovincialis*): The effect of natural dissolved organic matter on copper toxicity in estuarine waters. Sci. Total Environ..

[42] Lorenzo J.I., Nieto O., Beiras R. (2002). Effect of humic acids on speciation and toxicity of copper to *Paracentrotus lividus* larvae in seawater. Aquat. Toxicol..

[43] Lorenzo J.I., Nieto O., Beiras R. (2006). Anodic stripping voltammetry measures copper bioavailability for sea urchin larvae in the presence of fulvic acids. Environ. Toxicol. Chem..

[44] Li Y., Yuan H., von dem Bussche A., Creighton M., Hurt R.H., Kane A.B., Gao H. (2013). Graphene microsheets enter cells through spontaneous membrane penetration at edge asperities and corner sites. Proc. Nat. Acad. Sci. USA.

[45] Ivask A., Juganson K., Bondarenko O., Mortimer M., Aruoja V., Kasemets K., Blinova I., Heinlaan M., Slaveykova V., Kahru A. (2014). Mechanisms of toxic action of Ag, ZnO and CuO nanoparticles to selected ecotoxicological test organisms and mammalian cells in vitro: A comparative review. Nanotoxicology.

[46] Solioz M. (2016). Copper oxidation state and mycobacterial infection. Mycobact. Dis..

[47] Valko M., Morris H., Cronin M.T.D. (2005). Metals, toxicity and oxidative stress. Curr. Med. Chem..

[48] Wang Z., von dem Bussche A., Kabadi P.K., Kane A.B., Hurt R.H. (2013). Biological and environmental transformations of copper-based nanomaterials. ACS Nano.

[49] Kukla S.P., Slobodskova V.V., Zhuravel E.V., Mazur A.A., Chelomin V.P. (2022). Exposure of adult sand dollars (*Scaphechinus mirabilis*) (Agassiz, 1864) to copper oxide nanoparticles induces gamete DNA damage. Environ. Sci. Pollut. Res. Int..

[50] Ragusa M.A., Nicosia A., Costa S., Cuttitta A., Gianguzza F. (2017). Metallothionein gene family in the sea urchin *Paracentrotus lividus*: Gene structure, differential expression and phylogenetic analysis. Int. J. Mol. Sci..

[51] Burić P., Jakšić Ž., Štajner L., Dutour Sikirić M., Jurašin D., Cascio C., Calzolai L., Lyons D.M. (2015). Effect of silver nanoparticles on Mediterranean sea urchin embryonal development is species specific and depends on moment of first exposure. Mar. Environ. Res..

[52] Burić P., Čarapar I., Pavičić-Hamer D., Kovačić I., Jurković L., Dutour Sikirić M., Domazet Jurašin D., Mikac N., Bačić N., Lyons D.M. (2023). Particle size modulates silver nanoparticle toxicity during embryogenesis of urchins *Arbacia lixula* and *Paracentrotus lividus*. Int. J. Mol. Sci..

[53] George S., Lin S., Ji Z., Thomas C., Li L., Mecklenburg M., Meng H., Wang X., Zhang H., Xia T. (2012). Surface defects on plate-shaped silver nanoparticles contribute to its hazard potential in a fish cell line and zebrafish embryos. ACS Nano.

[54] Gilbertson L.M., Albalghiti E.M., Fishman Z.S., Perreault F., Corredor C., Posner J.D., Elimelech M., Pfefferle L.D., Zimmerman J.B. (2016). Shape-dependent surface reactivity and antimicrobial activity of nano-cupric oxide. Environ. Sci. Technol..

[55] Sartori D., Pellegrini D., Gaion A. (2016). Analysis of variability in embryological response of two sea urchin species to spatial and temporal features—Can these factors influence responses in standardized ecotoxicological assays?. Expert Opin. Environ. Biol..

[56] Masullo T., Biondo G., Natale M.D., Tagliavia M., Bennici C.D., Musco M., Ragusa M.A., Costa S., Cuttitta A., Nicosia A. (2021). Gene expression changes after parental exposure to metals in the sea urchin affect timing of genetic programme of embryo development. Biology.

[57] Pelikan J., Majnarić N., Maurić Maljković M., Pikelj K., Hamer B. (2022). Physico-Chemical and Ecotoxicological Evaluation of Marine Sediments Contamination: A Case Study of Rovinj Coastal Area, NE Adriatic Sea, Croatia. Toxics.

